# Annotations

**Published:** 1908-02-22

**Authors:** 


### ANNOTATIONS.

### Anæsthetic Fatalities.

At a meeting of the Medico-Legal Society, on February 11, a paper was read by Dr. L. Freyberger on "Deaths under Anæsthetics," the discussion on which has attracted some attention in the lay press. The paper contained a survey of some seventy-four anæsthetic fatalities investigated by Dr. Freyberger pathologically for Mr. Troutbeck, Coroner for Westminster and South-West London. The former was content to recite a catalogue of the cases with brief details of each, leaving the lessons and deductions therefrom to be drawn by subsequent speakers. Dr. F. W. Hewitt thought the series brought out a lesson which he has emphasised before—namely, that it is not the patient, not the drug, not the operation that chiefly affects a successful administration, but the personality of the anæsthetist. His other chief contention, that it is respiratory failure, whether due to obstruction of the air passages or not, which is the danger most to be feared and guarded against, was not supported by Dr. Leonard Hill, whose researches upon cats, and more especially upon dogs, led him to attribute an important *rôle* to direct action by the drug upon the heart. Dr. Hill is convinced that in apparatus for measuring and regulating the percentage of chloroform in the inspired air lies the hope of safety during anæsthesia. Subsequent speakers dwelt on the number of large hospitals in the South-West district, and commented on the small proportion of inquests on cases in private practice (3 in 74). Mr. Troutbeck thought the phrase "unnatural death" is probably more strictly construed in the hospitals than in private. On the motion of Dr. F. J. Smith, the debate stands adjourned to the next meeting of the Society, when it is to be hoped some member will explain, for the benefit of the non-medical members of the Society, the somewhat technical matters and methods in which the discussion has hitherto shaped itself.

### Serum Treatment.

In an article in the *Medical Chronicle*, Dr. Miles B. Arnold deals with some phases of serum treatment. He draws attention to the difference between antitoxic and bactericidal sera. Antidiphtheritic serum, belonging to the former group, is thus comparatively useless except in the early stages of the disease. Antistreptococcic serum, on the other hand, since it is bactericidal in action, should be tried as long as the specific organisms are believed to be free in the tissues, and as long as there is hope that no irretrievable damage has been done. Dr. Arnold agrees with the now generally accepted view that this serum, as well as others, should always be administered subcutaneously. As to the possible dangers of serum therapy, he states emphatically that the risk from a single injection is negligible, and should never be regarded as a contra-indication to the therapeutic use of serum, although repeated doses may cause much distress. In his *resumé* of the effects of serum injections, Dr. Arnold says that immediate somnolence, followed by temporary polyuria, is often present. The "reaction" occurs generally between the fifth and eleventh days. The rash may be of very short duration, an hour or less; and it may last for two or more days. It may be erythematous, urticarial, scarlatiniform, or morbilliform, or it may pass from one type to another. There is often severe itching, there is usually fever, but there is seldom malaise. Swelling and pains in the joints are less common but more troublesome; they may be migratory. The reaction has shown a decrease in frequency and severity during the last four years. Dr. Arnold is sceptical of the value of calcium salts as a prophylactic, since, according to the observations of Baginsky, the coagulability of the blood is actually increased in diphtheria.

### The Colouring of Milk.

Natural milk is white; after it has stood for some time the cream rises to the surface in a yellowish layer, but in no case is pure milk yellow all through. It might seem unnecessary to state such an obvious fact if it were not that the public is given to buying milk that looks "creamy," without considering that real cream does not affect the colour of the milk throughout its whole mass. The creamy tint, which sometimes almost reaches that of saffron, comes in most cases from the addition of colouring matter; it never makes the milk any better, while it may conceivably make it very much worse. One of the least parts of the mischief it does is that it enables a man to sell skimmed milk, denuded of its natural cream, as a high grade of new milk, and to compete unjustly with his honester neighbour, who sells his milk as it comes from the cow. This form of adulteration is practised most in the poorer districts, where the people are ignorant and easily imposed upon. Even when the colouring matters employed are not in themselves harmful, the fact of adulteration remains; but often the colour is given by coal tar products, which are in themselves undesirable. Besides being coloured, town milk is often "preserved," that is, boric acid or the like is put in to prevent it going sour, especially in hot weather. A small quantity of this may not be actively injurious, but those who use it are often not too careful as to the amount put in. A medical man who has examined specimens of milk from dairies in both well-to-do and poor neighbourhoods says that there are far fewer bacteria to be found in the milk taken from the latter; but adds that this proves no superior purity, but only the greater amount of preservative added to the milk. In spite of this absence of bacteria, we find summer diarrhœa most prevalent among children in the poor districts of our towns. While doubtless many factors tend to lower the health of these children, it seems as if non-bacterial milk did little to maintain their strength. But possibly what passes as diarrhœa is really an irritant poisoning of the stomach and intestines, due to the chemicals added to milk to improve its colour and prevent its souring. Pure milk is such an important element in our food, especially in that of children, that surely it should be compulsory under penalty for farmers or dealers to state when they put in colouring or preservatives.

